# Chemical variability of artificial stone powders in relation to their health effects

**DOI:** 10.1038/s41598-019-42238-2

**Published:** 2019-04-25

**Authors:** Francesco Di Benedetto, Andrea Giaccherini, Giordano Montegrossi, Luca A. Pardi, Alfonso Zoleo, Ferdinando Capolupo, Massimo Innocenti, Giovanni O. Lepore, Francesco d’Acapito, Fabio Capacci, Carla Poli, Tonina Enza Iaia, Antonella Buccianti, Maurizio Romanelli

**Affiliations:** 1 Dipartimento di Scienze della Terra, Università degli Studi di Firenze, via La Pira 4, Firenze, Italy; 2 INSTM Research Unit of Florence, Firenze, Italy; 3 CNR – Istituto di Geoscienze e Georisorse, via La Pira 4, Firenze, Italy; 4 CNR – Istituto dei Processi Chimico Fisici, via Moruzzi 1, Pisa, Italy; 5 Dipartimento di Scienze Chimiche – Università di Padova, Via Marzolo 1, Padova, Italy; 6 Dipartimento di Chimica, Università degli Studi di Firenze, via della Lastruccia 3, Sesto Fiorentino, Italy; 7 CNR – Istituto di Officina dei Materiali - OGG c/o, ESRF, Grenoble, France; 8 Dipartimento di Prevenzione, PISLL, Health Agency of Tuscany (USL Toscana Centro), Tuscany, Italy

**Keywords:** Environmental sciences, Health occupations

## Abstract

The occurrence of highly severe silica-related diseases among the resin- and silica-based artificial stone workers was claimed, associated to an extremely short latency. High levels of exposure and intrinsic properties of AS are thought to modulate the development of silicosis and auto-immune diseases. This study compares parent materials and processed dusts, to shed light on changes of AS occurring in the manufacturing process, through an XRF, EPR and XAS investigation. We point out the extremely wide variability of the materials, the occurrence of chemical signatures impressed by the processing techniques, and the unprecedented generation of stable radicals associated to the lysis of the Si-O chemical bond inside the resin coated respirable crystalline silica. These results suggest that the AS processing in industrial stone workshops can create respirable dusts with peculiar physical and chemical properties, to be correlated to the observed clinical evidences.

## Introduction

Artificial stone (AS) is a composite material realised assembling powders, and occasionally fragments, of natural stones with a binder^1–5^. Recent kinds of AS mainly employ unsaturated polyester resins^6^ as binder^7,8^, the filler eventually being waste material from the manufacture of natural stone^9^. These ASs exhibit good technological features and they are generally used to realise kitchen and bathroom countertops^10^. Two main types of AS are present on the market, involving either carbonates (marble, travertine)^6–9^, or silica^1,11,12^. Other natural or synthetic mineral phases are included to confer to the final stone specific appearances^9^ or technological properties (e.g. porosity, hydrophobicity, …)^7,8^.

After the inlet of resins- and silica-based AS in the large-scale production (in 1986^1^), the occurrence of silica-related diseases among the AS workers (ASW) was claimed^11–13^. Then, some relevant crops of silicosis were reported^1,10,13–17^. The latency of the silicosis in ASW is extremely short (~10 years), and the severity of the diseases is high^10,14,15^. Exposure to AS was also linked to severe auto-immune diseases in ASW^16,18^. Usually, such severity is associated to the lack of adequate preventive actions^19,20^. However, there’s a wide consensus that the high levels of exposure to silica dust can hardly justify alone all clinical findings^1,19^. Hoy *et al*.^1^ pointed out specific effects likely linked to “peak level exposure”, more severe than the average one, seldom reached during a 8-hours working day.

The study of Pavan *et al*.^19^ represents, to the authors’ knowledge, the unique attempt to link the health effects on ASW to specific physico-chemical and morphological features of the processed materials. Specifically, these authors suggested the occurrence of a film of resin coating the respirable crystalline silica (RCS) particles. Resin coating could affect all the reactive pathways of the particle surfaces, and it could set up a complex interaction with the lung lining fluids. A relevant role by redox active species in the materials (e.g. Fe, Cu, …) was also inferred.

The present experimental study compares parent materials and processed dusts, obtained from different production lines, to shed light on changes of AS through the manufacturing process.

## Materials and Methods

### Investigated samples

Materials were sampled at three different industrial stone workshops. Seven different commercial artificial stones were selected because of their different characteristics (mainly colour and fabric). Raw fragments of AS are labelled as #C (Table 1 and Supporting Information, SI, Section A). Two other materials for each AS were sampled: dusts (#B) from conventional mechanic treatments operated under dry conditions; dusts (#A) from mechanic treatments operated under wet conditions (i.e. abated by water). This set of samples includes part of the samples investigated by Pavan *et al*.^19^.

**Table 1:** List of investigated samples.

Factory	Raw	Dry polishing	Wet cut
Workshop 1	1C, 2C, 4C	1B, 2B, 4B	1A, 2A, 4A
Workshop 2	3C	3B	3A
Workshop 3	5C, 6C, 7C	5B, 6B, 7B	5A, 6A, 7A

### Sample characterization and statistical analysis

Mineralogical speciation and chemical composition of samples were investigated through Scanning Electron Microscopy (SEM), X-ray Powder Diffraction (XRPD), X-ray Fluorescence (XRF), X-ray Absorption Spectroscopy (XAS)^21,22^ and continuous wave (cw) and pulsed Electron Paramagnetic Resonance (EPR) spectroscopy. Further experimental details are summarised in the SI (Section B). The XRF chemical data were analysed by means of statistical approaches, including univariate boxplots and multivariate analysis (cluster analysis and Principal Component Analysis). The compositional data were transformed by using the log-centred transformation^23^, to avoid mathematical constraints and biased interpretation of the natural relationships^24,25^. Further details are summarised in the SI (Section C).

## Results

### Mineralogical characterisation

All samples consist mostly of quartz, with very few, when detectable, associated phases, in good agreement with the literature^19^. The unique noticeable difference concerns the mineralogical composition of the sample of the series 4, characterised by the massive presence of cristobalite. Further details are reported in SI (Section D).

### Chemical composition of the samples

**Figure 1:**
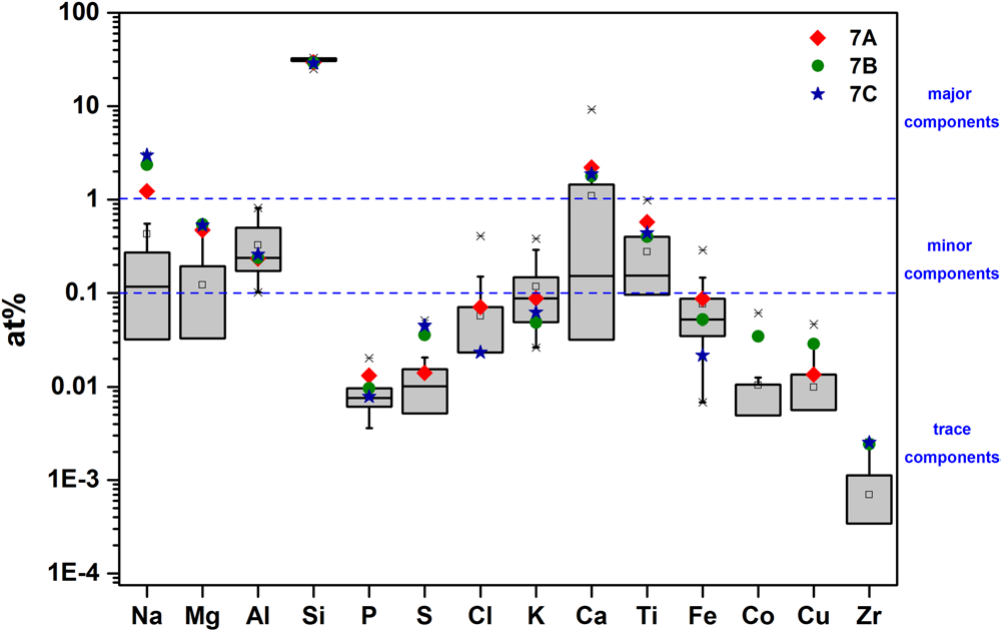
Boxplots of the elemental composition of the samples. The blue stars, green circles and red diamonds represent the analytical results for the 7C, 7B and 7A samples, respectively.

The sample chemical composition, investigated through XRF, is largely dominated by the silica content. Moreover, samples exhibit a significant chemical variability, testified by the ranges reported in Table 2 and SI (section E) and by the boxplot (Fig. 1). Assuming conventional limits to discriminate major, minor and trace elements (i.e. 1% and 0.1%), one can observe that only Na and Ca are seldom occurring as major elements, in agreement with the AS mineralogical composition. Among minor elements, Na, Mg, Al, K, Ca, Ti are frequently found, while Cl and Fe show seldom occurrences. Na, Mg, K and Ca exhibit ranges fully covering both the trace and minor element fields, whereas P, S, Cl, Fe, Co, Cu and Zr are trace contaminants. The elements not shown in Fig. 1 never exceed the field of the minor elements, being mostly trace contaminants. Zn occurs as minor component only in three samples (7A, 7B and 7C). Moreover, data concerning some other elements (Na, Mg, Ca and Ti, and, partly, P, S, Co and Cu) suggest that the chemistry of these three samples is markedly different from that of all other analysed samples (Fig. 1). Since their anomalous behaviour can affect the join multivariate structure of the data^26,27^ the subsequent multivariate analysis was performed without including them in the database (SI, Section E).

**Table 2:** Number of cases, maximum, minimum and median at% values from XRF analyses.

Element	N	Max	Min	Median
*Na*	17	2.982	0.017	0.209
*Mg*	13	0.542	0.017	0.109
*Al*	21	0.814	0.102	0.237
*Si*	21	32.97	25.03	32.15
*P*	18	0.020	0.004	0.008
*S*	20	0.051	0.003	0.010
*Cl*	11	0.407	0.023	0.071
*K*	21	0.380	0.026	0.087
*Ca*	20	9.200	0.014	0.166
*Ti*	20	0.981	0.011	0.158
*Cr*	8	0.014	0.004	0.009
*Mn*	2	0.009	0.006	0.007
*Fe*	21	0.288	0.007	0.052
*Co*	12	0.061	0.005	0.009
*Ni*	5	0.005	0.002	0.003
*Cu*	13	0.046	0.004	0.010
*Zn*	3	0.014	0.010	0.012
*Sr*	5	0.006	0.0007	0.001
*Zr*	10	0.002	0.0007	0.001
*Sn*	4	0.007	0.003	0.006
*Ba*	2	0.006	0.005	0.006
*O*	21	66.73	63.73	66.60

**Figure 2:**
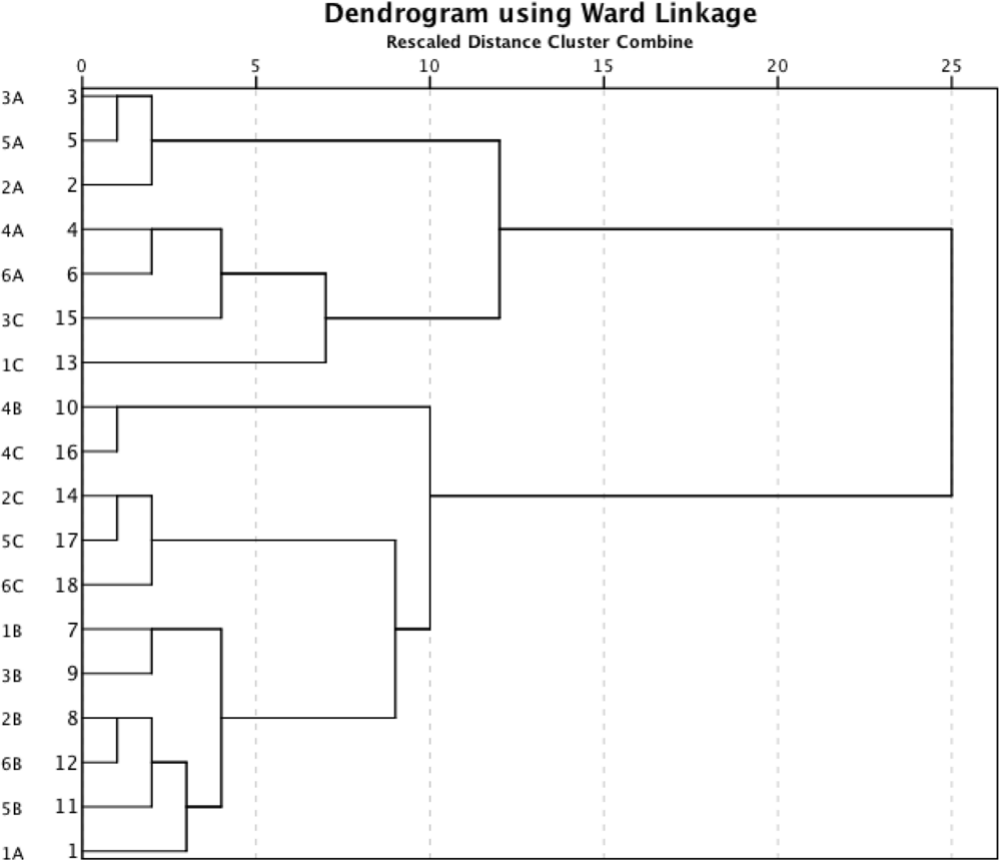
Dendrogram of the XRF compositional dataset.

The dendrogram (Fig. 2) points to two main groups of samples. A and B samples cluster in different groups (with the exception of the 1A sample), whereas C samples do not exhibit preferential grouping (with A or B) nor group alone. Considering that C samples are parent of both A and B, these latter being linked to different stone processing, the dendrogram points to the occurrence of a certain “chemical signature” impressed by the processing. The cluster analysis was performed on log-centred data by using the squared Euclidean distance as similarity measure and the Ward method to link cases^28^, (SI section C).

**Figure 3:**
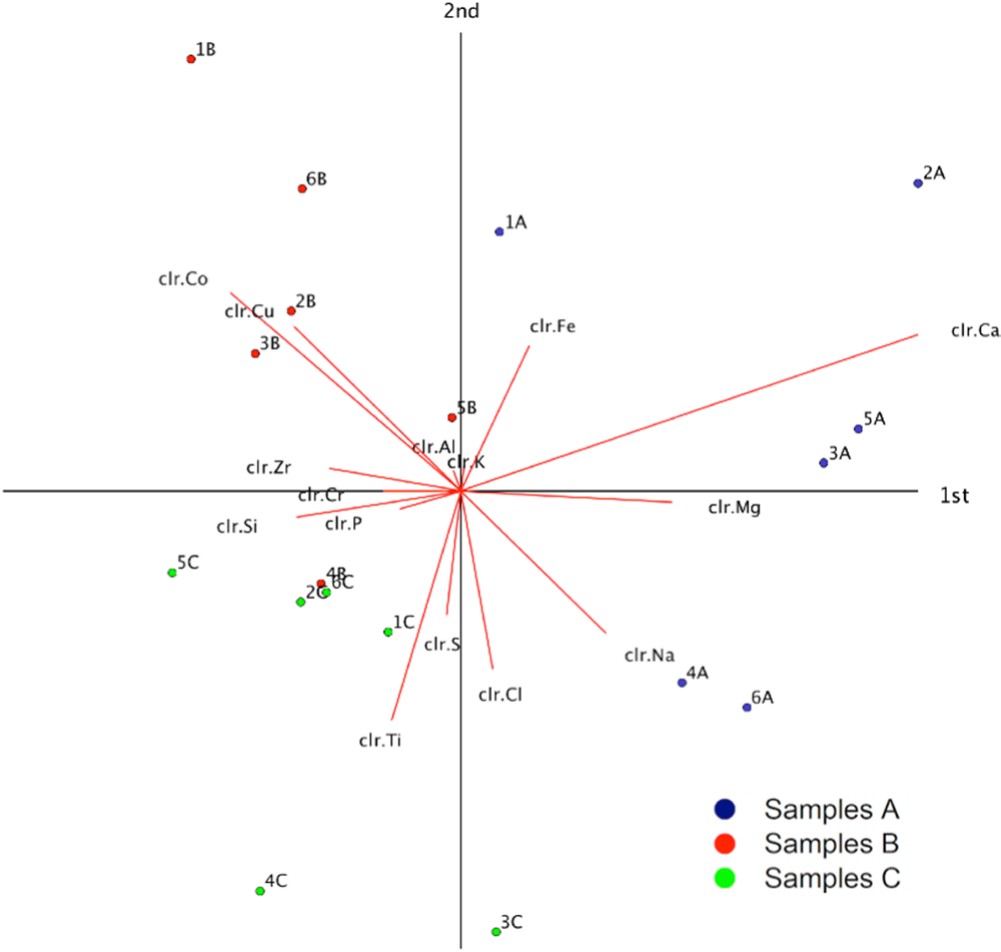
Biplot of the XRF compositional dataset. The axes define the direction of maximal variability of the n-dimensional constrained space of the database. In blue, red and green are indicated results pertaining to the A, B and C sample series, respectively. The red line direction indicate the localisation of the contribution given by a single element, whereas the red line length is linked to its variance.

A further step in the analysis of the dataset variability has been carried out using the biplot methodology for compositional data^24,29^ (SI, sec. C). Having the longest rays (Fig. 3), Ca, Na, Co and Ti explain most of the variability of the XRF dataset, with respect to the compositional barycentre. These rays define quadrants where samples of different groups are well discriminated. In particular, the rays of Na and Ca (plus Mg, Cl, Fe) head towards samples of groups A, whereas rays of Co (plus Cu, Zr, Cr, Al, K, Fe) towards group B and Ti (plus Si, P, S, Cl) towards group C. Indeed, the A, B and C groups appear characterised by a different compositional signature. Closer detail on the relationships among elements in the biplot has been provided by opportune sets of 3 variables (sub-compositions), representable in a ternary diagram (SI, section F). In the following, two specific hypotheses about the origin of contamination of samples during processing have been tested exploiting specific ternary diagrams:

- Contamination occurring at the interface between water and CS (wet processing)
- Contamination occurring at the interface between CS and machine- tool materials (dry processing)

**Figure 4:**
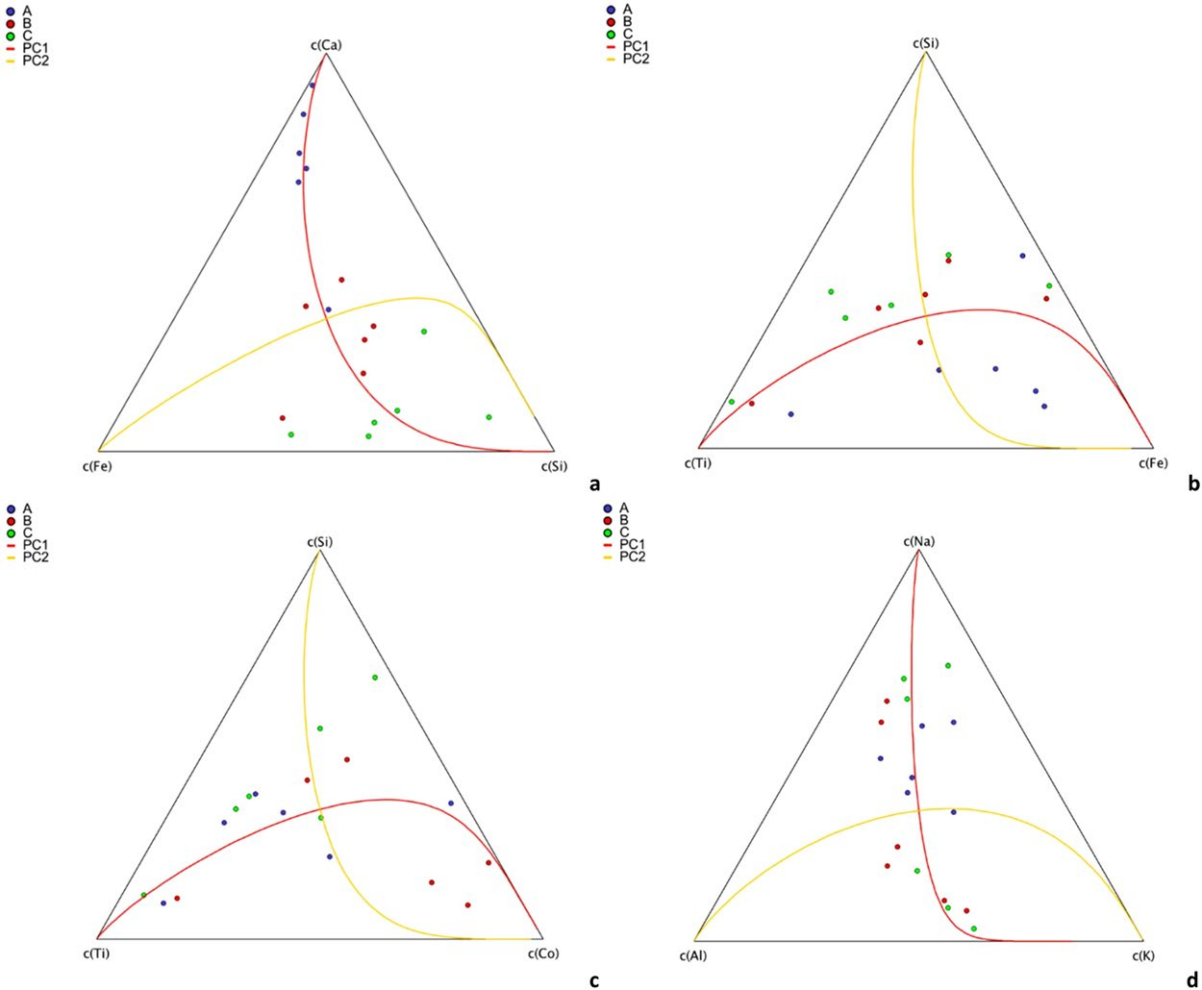
Ternary diagrams of sub-compositions: (**a**) Ca-Fe-Si; (**b**) Si-Ti-Fe; (**c**) Si-Ti-Co; (**d**) Na-Al-K. In blue, red and green are indicated results pertaining to the A, B and C sample series, respectively. The two continuous line represent the eigenvectors of the ternary sub-compositional space.

The first hypothesis was tested through the study of the subcomposition Ca-Fe-Si. The ternary diagram (Fig. 4a) points to an increase in Ca associated with an increase in the ratio Fe/Si, as shown by the PC1 direction, which explains ~90% of the data variability. Accordingly, the internal relationship among these three variables appears a feature of the whole system. Thus, the diagram of Fig. 4a supports the fact that a part of Fe is added to the system together with Ca by wet processing.

Concerning the second hypothesis, the dry process may result in a sample contamination accounting for the relative hardness of the minerals/materials involved. Under this scheme, Ti is contributed by rutile, Fe by tools and Si by CS. The diagram of the Si-Ti-Fe sub-composition (Fig. 4b), the PC1 of which explains ~83% of the data variability, points to a light increase in Si associated with a high variability in the Fe/Ti ratio. These results indicate an alternative presence of either Ti or Fe, in agreement with the hardnesses of rutile and steel, respectively. The higher the rutile content, at the expenses of the CS content, the softer is the material, and thus the Fe content decreases. In order to assess to which extent Fe can be considered a proxy of the contamination by working-tools, we used an indirect method, considering Co as an element of special steel. The ternary diagram of the sub-composition Si-Ti-Co (Fig. 4c), with PC1 capturing the 82% of the data variability, reveals a close similarity to that of Fig. 4b. Thus, we can safely assume that at least a part of the Fe on the processed samples is provided as an impurity during the processing step, and that the amounts provided by the wet and dry processes may not be characterised by an equivalent speciation.

Na is one of the most variable elements in the compositional dataset, suggesting possible contributions by different sources (Fig. 3). Figure 4d depicts the ternary diagram of the Na-Al-K field, chosen to assess if the association between these three elements (commonly found associated in alkaline feldspars) was occurring. The observed trend confirms that Al and K, whose ratio is almost constant, can be attributed, as hypothesized, to feldspars, contained in the parent materials (in agreement with the XRD findings). Conversely, Na is provided by at least two sources, the most relevant of which is not linked to the parent materials, and it is likely associated to the wet processing.

### EPR spectroscopy: transition metal ions

**Figure 5:**
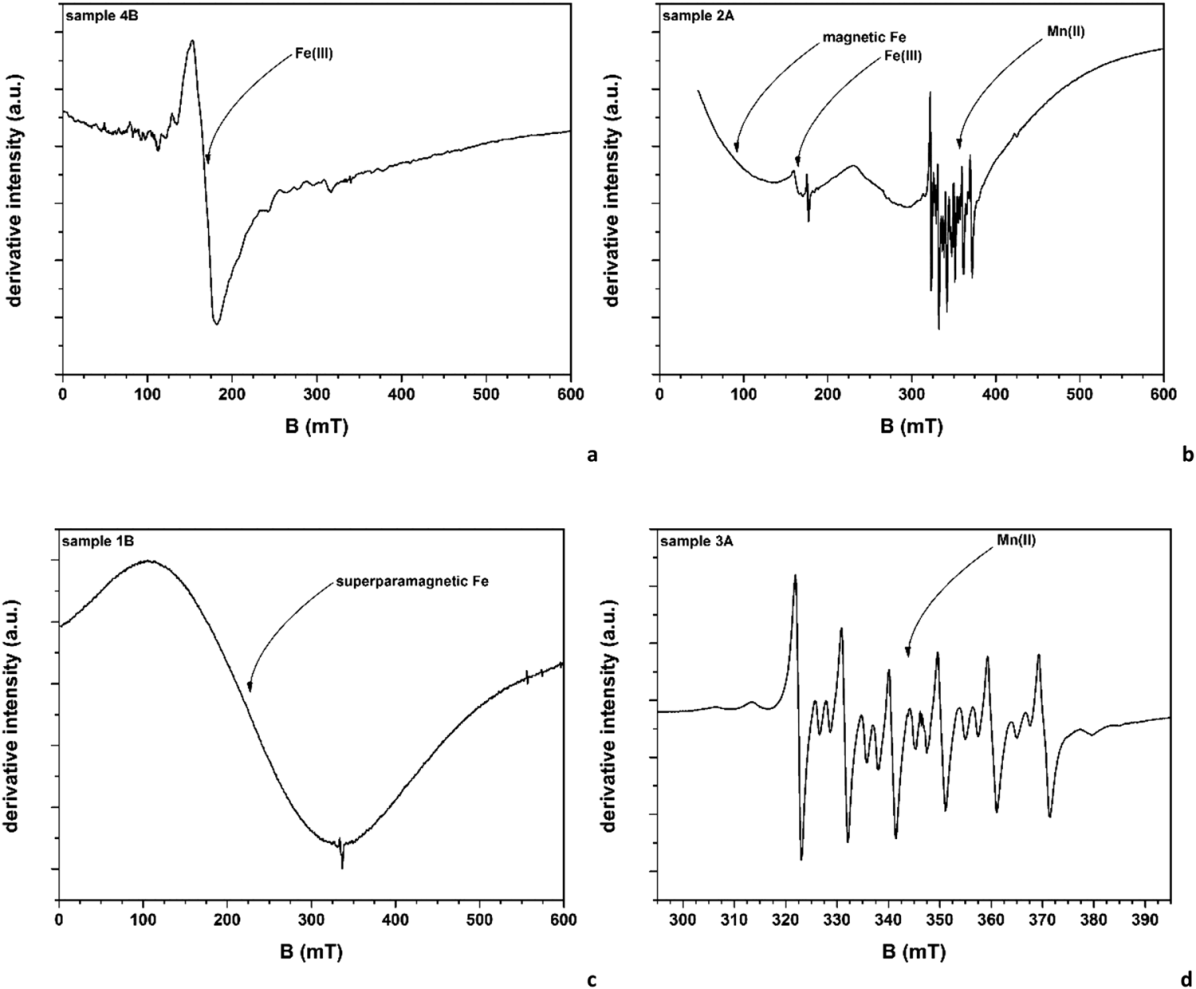
Selected representative EPR spectra of species discussed in the text. Spectra plot the first derivative of the signal intensity versus the applied magnetic field (expressed in milliTesla, mT).

The intrinsic heterogeneity of the samples, and especially the variability of the chemical composition, has a counterpart in the results of the EPR investigations. Considering only the analytically detected elements, at least Al, Si, Ti, Cr, Mn, Fe, Co, Cu can occur as EPR active species (transition metal ions, TMI, and/or inorganic radicals). The spectral variability, observed in the panoramic EPR spectra of all 21 samples (in SI, Section G), can be ascribed to this circumstance. However, most of the main experimental features can be attributed to the Fe speciation. Signals from three different Fe species have been observed: (a) Fe(III) as isolated ion, occurring in a rhombic coordination, revealed by the typical narrow EPR signal at g ~ 4.3 (B ~ 165 mT), as in Fig. 5a; (b) Fe occurring in a permanently magnetized phase (such as metallic Fe, magnetite, hematite, …), as in the case of Fig. 5b: in this case, the extremely broad width, and the occurrence of a zero-field absorption, unambiguously indicate the excitation of magnetic resonant modes^30^; (c) Fe occurring in superparamagnetic particles, whose crystal size is small enough to behave as single-domain magnetic particles, as in the case of Fig. 5c; these species are characterised by moderately broad signals (ΔH < 200 mT), whose centre changes towards lower magnetic field values with decreasing temperature^30^. Only the EPR signal of Mn(II), among other TMI possibly occurring in the samples, has been frequently observed (Fig. 5d). This species, with its isotropic hyperfine structure, is likely to occur in a carbonate environment^31^. The overall findings of the EPR investigation are summarised in the Table 3.

**Table 3:** Summary of the species identified by EPR (Ab-abundant; Ra-rare).

	Magnetic Fe species			Superparam. Fe species			Fe(III)			Other TMI		
	C	A	B	C	A	B	C	A	B	C	A	B
1	—	—	—	Ab	Ab	Ab	—	—	—	—	—	—
2	—	Ab	—	Ab	—	Ab	—	Ra	—	—	Mn(II)	—
3	—	—	Ab	Ra	—	—	—	Ra	—	—	Mn(II)	Mn(II)
4	—	Ab	Ab	Ra	Ab	—	Ra	Ra	Ab	—	Mn(II)	Mn(II)
5	—	Ab	—	Ab	Ab	Ab	—	—	—	—	Mn(II)	—
6	—	Ra	Ra	Ra	Ra	—	Ra	—	—	—	Mn(II)	—
7	—	Ra	Ab	Ra	Ra	—	Ra	—	—	—	Mn(II)	—

Certain Fe species apparently show a general trend over the set of investigated samples. The most striking trend concerns the permanent magnetic species. These, in fact, are detected in A and B, but not in C samples. This suggests a link with the treatments undergone by the A and B samples: worked materials are contaminated by magnetic Fe species during the cutting processes. Conversely, no evidence of a significant discrimination among the A and B samples was observed. Signals attributed to superparamagnetic Fe species are observed in all C samples, and in most of the treated A and B samples, without evidence of specific trends. However, the data in Table 3 suggest that the superparamagnetic species, when abundantly present in the C samples, are always found also in the treated samples. This fact could be interpreted considering these species as isolated from the silica framework, and, being softer^32^, transferred to the treated dusts. Fe(III) ions are only seldom observed. Mn(II) is never detected in the raw C samples, while being preferentially found in the wet A samples. The fact that A samples are characterized by a robust enrichment in Ca(II) (see §3.2) and the attribution of the Mn(II) spectrum to calcium carbonate, point to a definite relationship between these two elements. We thus infer that Mn(II) contamination occurs, during the wet cutting, through the water used in abating procedures.

### EPR spectroscopy: radical species

**Figure 6:**
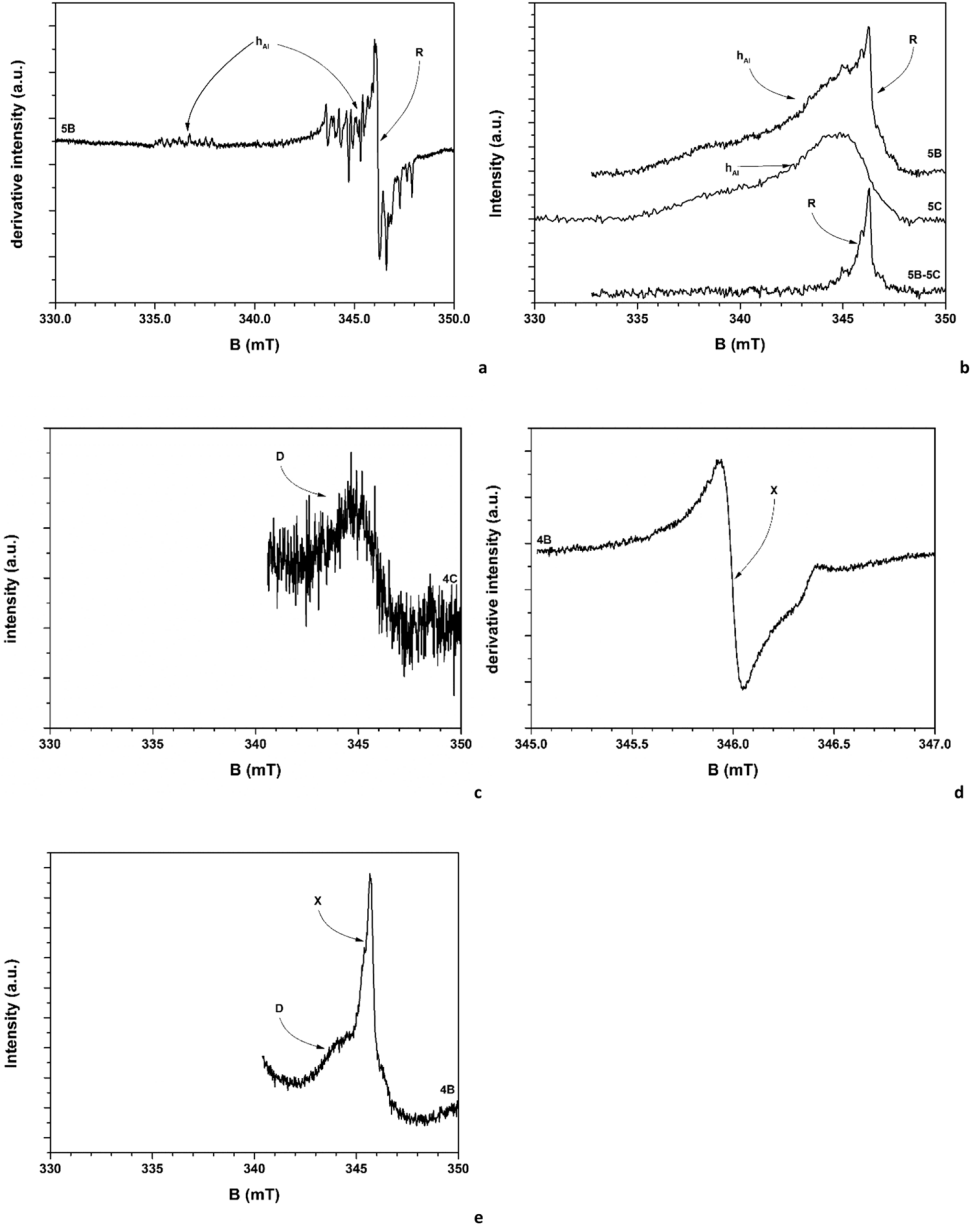
Detail EPR (**a,d**) and EEPR (**b,c,e**) spectra of the radical species. EPR spectra plot the first derivative of the signal intensity versus the applied magnetic field (expressed in milliTesla, mT), whereas EEPR spectra plot the signal intensity versus the applied magnetic field (expressed in milliTesla, mT).

All samples reveal a noticeable spectrum due to the h_Al_ radical^33^. This spectrum consists of two multiplets, centred at B = 337 mT and at B = 346 mT, due to the superhyperfine interaction of the unpaired electron, located onto an O^−^ anion, with neighbouring H and Al nuclei (Fig. 6a). This radiogenic radical, very common in quartz, is located inside the crystals, with the proton hosted in the channels parallel to [001] axis in the α-quartz structure. The presence of the h_Al_ spectrum is observed almost independently on the type of process followed by the investigated A and B samples. Thus, it cannot be considered as an efficient proxy of changes in the radical speciation during AS processing. It is worth mentioning that no h_Al_ signal is detected in the samples of the series 4, in full agreement with the almost complete absence of quartz (§3.1 and SI, Section H).

All A and B samples, but those of the series 4, present the ubiquitary evidence of a further radical species, hereafter called as R, superimposed to the h_Al_ spectrum. Its presence, however, is never observed in the #C samples. Thus, the R radical can be considered as a product of the AS processing. From the detailed EPR spectra (e.g. Fig. 6a), only an approximate definition of its parameters can be gained, i.e. its position, corresponding to a value of g = 2.0032(2) and its very narrow width, of the order of 0.15 mT. No evidence of hyperfine structure of the signal is observed. A closer detail of the R species was obtained through the EEPR investigation: from this technique, the experimental spectra of both the 5B and 5C samples reveal, as previously stated, the presence of the asymmetric spectrum of the h_Al_ radical (the hyperfine structure of this species is unresolved due to the used echo sequence^34^, Fig. 6b). The spectrum of the 5B sample presents a further signal, due to the R species. The simultaneous presence of both spectra points to the attribution of the R radical to a species with the same S = 1/2 spin state as the h_Al_ centre. A better visualization of the signal due to the R species was obtained by subtracting the spectrum of the 5C sample from that of the 5B one (Fig. 6b). The residual signal intensity is thus fully attributable to the R species. A spectral fit carried out assuming a Lorentzian line shape indicates that the spectrum is characterized by a slight anisotropy in the g values (2.0043(2) and 2.0032(2) for the parallel and perpendicular components, respectively) associated to a larger difference in the line width (1.04(3) and 0.20(1) mT, respectively). The absence of hyperfine interaction was confirmed. On this basis, we can tentatively attribute this signal to an unpaired electron not chemically bonded to magnetic nuclei, with g values compatible with either an inorganic radical as e.g. the E centre in quartz (i.e. an unpaired electron localized on a tri-coordinated Si atom)^35^, or to an organic radical, likely generated during the thermal and mechanical of sample processing.

The radical speciation of the samples belonging to the series 4 appears to be different. In the 4C sample, a very broad and undefined signal at g ≈ 2 (labelled as “D”) is registered. However, the very bad signal-to-noise ratio prevents any kind of more accurate characterization of its position and shape. Despite the bad spectral quality, the EEPR spectrum of the D species (Fig. 6c) reveals a slightly broad signal, centred at g = 2.0040(2), and a line width of 1.9(1) mT. The very poor signal-to-noise ratio allows its attribution to a radical species, originally present in the polyester resin; the absence of h_Al_ radicals is in fact linked to the absence of quartz. This attribution is also in agreement with the fact that cristobalite, a high temperature polymorph of silica, crystallizes without intrinsic radicals^35^. When sample 4C undergoes processing, EPR (Fig. 6d) and EEPR (Fig. 6e) indicate that a new radical species is formed (labelled as “X”). This species is observed on both 4A and 4B samples. In fact, the EEPR of Fig. 6e shows that the sharp line of the species X is overlapped on the broader line of the D species. The X line position is consistent with a g = 2.0030(2) value, very similar to that of the R radical. However, the absence of any appreciable Zeeman anisotropy is supporting the attribution to a different species. The line width of the spectrum of the radical X is 0.45(2) mT. This result suggests further considerations. As far as the radical speciation is concerned, the quartz-bearing and quartz-free samples subjected to processing exhibit different results, so the final radical speciation is closely linked to the mineralogical composition of the original material. In contrast, no, or very weak, apparent relationships are found with the sample chemistry (i.e. with respect to the chemical signatures described §3.2) or in the type (wet, dry) of processing. Moreover, the resin in its original state may contain some radicals, but these are very few, especially if compared with h_Al_, whose concentration has been estimated in 5*10^14^ defect/mol^33^, and they seem substantially unaffected by the sample processing. Thus, we attribute the newly formed R and X species to the interaction between the surfaces of mineral/resins and of processing tools.

### ESEEM spectroscopy

**Figure 7:**
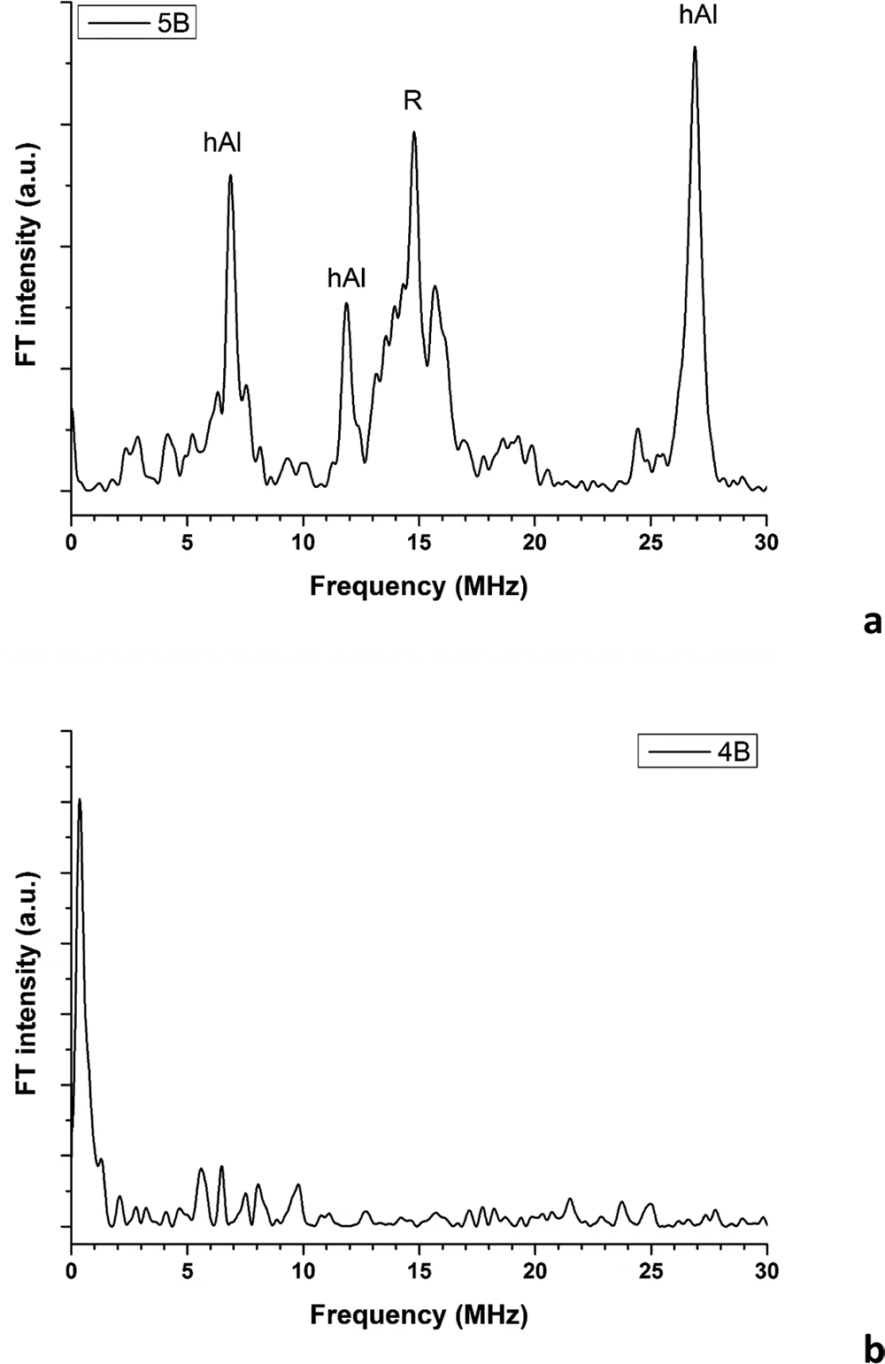
Exemplar patterns of the FT intensity versus the frequency (expressed in MHz) of (**a**) 5B and (**b**) 4B samples.

Exemplar ESEEM Fourier Transform (FT) patterns are shown in the Fig. 7 (all FT patterns are shown in SI, Section I). Apparently, all A and B samples but those of the series #4 present a common FT pattern (Fig. 7a): they mark the presence of different groups of peaks. Those at ~7–9, ~11–12 and ~23–27 MHz are due to the interaction of the h_Al_ radical with the neighbouring H nuclei, within the channel of the quartz structure^33^. Conversely, the additional peak at ~14–15 MHz can be attributed to the Larmor resonance of free H nuclei, i.e. to several nuclei that weakly interact with the paramagnetic centre through dipolar interaction. This signal is attributed to the R species. If hosted inside the quartz crystal, or inside the resin, the R species would exhibit strong dipolar and probably isotropic hyperfine interactions: thus, we can reasonably state that the R species is located at the interface between the resin and the crystal, i.e. it is a surface species, of the type of the E centres^35^.

Concerning the FT spectra of the 4A and 4B samples, no appreciable nuclear (proton) modulation patterns are observed (Fig. 7b). Accordingly, also the X radical does not belong to the resin. However, no information about the local surrounding of the X species was gained. Thus, considering the main species present in the 4B and 4C samples, i.e. cristobalite, rutile, and resin, we can exclude only the last one. The two tentative attributions can be surface radicals of either cristobalite, or rutile. One has to consider, that for this second attribution, the weak peaks in the FT pattern of Fig. 7b, occurring at 0.35 MHz and 1.30 MHz could be attributed to hyperfine interaction with ^47^Ti and ^49^Ti isotopes, with quadrupolar contribution.

### X-ray absorption spectroscopy

The analysis of the XANES and EXAFS regions highlights that Fe is present in multiple species in all the studied samples. The co-presence of different oxidized phases, together with metallic Fe, was therefore always taken into account. Linear combination fits (LCF) of the XANES region were performed employing several Fe-bearing minerals as standards (Section J in SI). The ubiquitous presence of Fe in multiple phases does not allow one to identify unambiguously all the host components and LCF results should be regarded as merely indicative of the “kind” of Fe-bearing phases.

Data from LCF of the XANES were then used as a starting point to sort out the most probable components contributing to the overall experimental EXAFS spectra. The quantitative analysis of the EXAFS data was carried out considering the possible co-presence of metallic Fe together with other oxidized phases. Whenever the presence of metallic Fe was highlighted by the LCF results, fits were performed by initially assigning two different amplitude factors to the “Fe-oxide” phase and metallic Fe. After the achievement of a suitable model for the oxidized phase, a single amplitude factor was used. The metallic and oxidized phases were then related to the overall amplitude by means of a proportional factor and their sum constrained to be 1, thus obtaining an estimation of the ratio between metallic and total Fe content in a manner similar to that described by Di Benedetto *et al*.^36^ (Table 4).

**Table 4:** Main parameters for EXAFS analysis.

	R factor	e_0_	S_0_^2^	Fe^0^ (molar fraction)	path	N	R (Å)	σ^2^ (Å^—2^)	D. T. (K)
1A	0.02	−10(1)	0.6(1)	0	Fe-O	6	1.98(1)	0.007(2)	
					Fe-Fe	8	2.96(2)	0.016(2)	
					Fe-Fe	4	3.01(2)	//	
					Fe-Fe	4	3.43(2)	//	
1B	0.02	7(1)	0.8(1)	0.3(1)	Fe-O	6	2.03(1)	0.010(4)	
					Fe-Fe	4	2.99(1)	0.005(3)	
					Fe^0^				354(86)/302(58)
1C	0.009	3(1)	1.1(1)	0	Fe-O	6	1.99(1)	0.015(3)	
					Fe-Fe	4	3.00(1)	0.012(2)	
					Fe-Fe	4	3.41(1)	//	
2A	0.02	−7(2)	0.6(1)	0	Fe-O	6	2.05(2)	0.007(4)	
					Fe-Fe	4	3.02(2)	0.013(4)	
2B	0.04	9(2)	0.9(1)	0.6(1)	Fe-O	6	2.04(1)	0.013(9)	
					Fe^0^				350(37)/312(27)
2C	0.002	−8(1)	0.7(1)	0	Fe-O		1.97(1)	0.004(1)	
					Fe-Fe		2.99(1)	0.010(1)	
					Fe-Fe		3.51(1)	//	
3A	0.01	−7(2)	0.6(1)	0	Fe-O	6	2.06(1)	0.006(3)	
3B	0.03	7(1)	0.6(1)	0.5(1)	Fe-O	6	2.07(1)	0.009(5)	
					Fe^0^				422(44)/368(28)
3C									
4A	0.03	7(1)	0.7(1)	0.5(2)	Fe-O	6	2.08(1)	0.012(7)	
					Fe^0^				389(49)/330(28)
4B	0.03	8(2)	1.0(3)	0.4(1)	Fe-O	6	1.99(1)	0.03(1)	
					Fe^0^				416(59)/361(38)
4C	0.02	−8(4)	0.8(2)	0	Fe-O	4	1.81(2)	0.003(3)	
5A	0.001	−6(1)	0.62(3)	0	Fe-O	6	2.03(1)	0.011(1)	
					Fe-Fe	2	2.99(1)	0.004(1)	
5B	0.02	4(1)	0.8(1)	0	Fe-O	6	1.99(1)	0.007(3)	
					Fe-Fe	2	2.93(1)	//	
					Fe-Fe	2	2.99(1)	0.003(2)	
					Fe-Fe	1	3.17(1)	//	
					Fe-Fe	1	3.43(1)	//	
5C	0.01	−10(1)	1.0(1)	0	Fe-O	6	2.01(1)	0.005(2)	
					Fe-Fe		2.92(1)	0.010(1)	
					Fe-Fe		2.99(1)	//	
					Fe-Fe		3.38(1)	//	
					Fe-Fe		4.58(2)	//	
6A	0.03	2(2)	1.0(2)	0.3(1)	Fe-O	6	2.07(1)	0.011(4)	
					Fe^0^				324(74)/253(41)
6B	0.04	0(2)	0.9(2)	0.11(4)	Fe-O	6	2.03(1)	0.012(4)	
					Fe-Fe		3.00(2)	0.018(6)	
					Fe^0^				324*/253*
6C	0.02	−2(2)	1.1(2)	0	Fe-O	6	2.03(2)	0.008(3)	
					Fe-Fe	1	3.00(5)	0.002(2)	
					Fe-Fe	1	3.35(5)	//	
7A	0.02	2(1)	0.6(1)	0.6(1)	Fe-O	6	2.06(2)	0.003(6)	
					Fe^0^				373(50)/346(35)
7B	0.04	3(2)	1.1(3)	0.5(2)	Fe-O	6	2.11(2)	0.02(1)	
					Fe^0^				285(64)/224(38)
7C	0.05	−3(2)	0.6(1)	0	Fe-O	4	1.93(1)	0.001	

Notes: S_0_^2^ = Amplitude reduction factor, R = refined path distance, N = path degeneracy, *σ*^2^ = Debye-Waller factor. * = Fixed parameters. Metallic Fe (Fe^0^) has been fitted according to the BCC structure using the correlated Debye model in order to compute Debye-Waller factors for each path^45^ and employing two different variables (D.T. = Debye Temperature): one for the I shell and one for the higher coordination shells. The average of multiple Fe-O distances is reported for the I shell.

**Figure 8:**
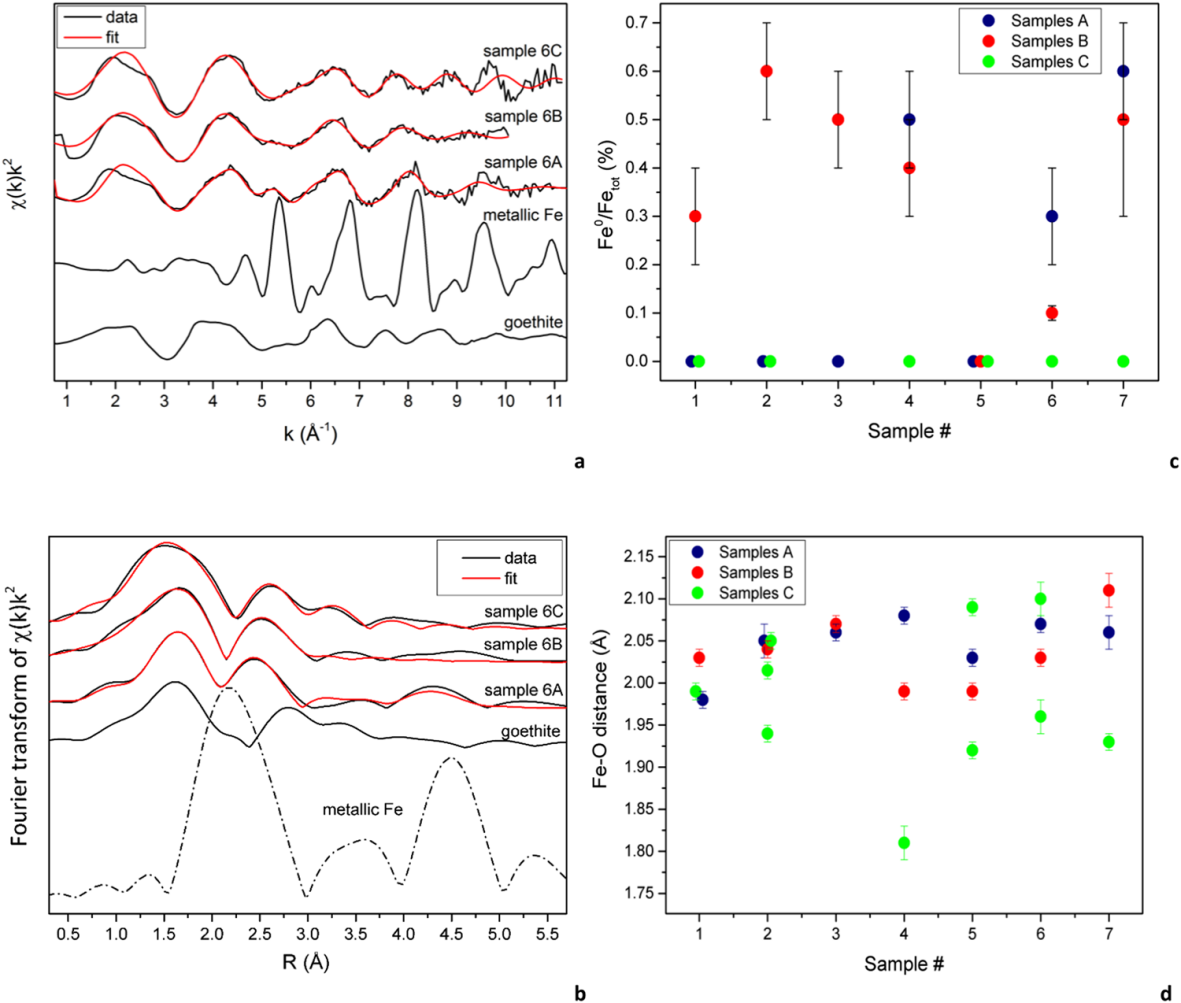
(**a**) EXAFS and (**b**) FT of samples 6A, 6B and 6C, together with multiparameter fit and reference compounds; (**c**) ratio between metallic and total Fe content (Fe^0^/Fe_tot_) evaluated from EXAFS multiparameter fit; (**d**) Fe-O bond distance (in Å) in studied samples.

The EXAFS signal of the samples #6A, shown as example in Fig. 8a,b, is dominated by an oxidized component; however, the comparison with the metallic Fe spectrum suggests the presence of moderate amounts of this species. Indeed, the quantitative EXAFS analysis yielded a metallic Fe fraction of about 30(10) % of the total Fe content with a double distance 2.48(3)–2.88(3) Å for metallic Fe, typical of the bulk bcc metal. Figure 8c shows the metallic Fe fraction thus obtained by the EXAFS analysis on all the studied samples; such values are in good agreement, especially if considering the complexity of the studied system, with those obtained by the LCF analysis. The most evident feature is that none of the raw samples shows the presence of metallic Fe (Fig. 8c), while the mechanically processed samples, both in wet and dry conditions, show in almost every series the presence of variable amounts of metallic Fe, with contents reaching up to the 60% of the total Fe content.

As for the oxidized portion of the spectrum, Fig. 8d shows the results obtained on the I-shell bond distance; in a few cases, the EXAFS analysis of the raw samples required the use of multiple paths in order to properly model the data. Second shell contributions are inconclusive since the presence of multiple Fe-bearing phases makes unambiguous information hard to infer. For the same reason we are prevented from getting information on the host phases by the investigation of the first shell only: indeed, Fe could be present in at least 4 different coordination environments, other than that pertaining to its metallic form, since the presence of both tetrahedral and octahedral Fe at both 2+ and 3+ oxidation states, cannot be excluded. However, the obtained data allow us to infer some information about the effects of the mechanical treatments. The Fe-O bond length of the treated samples rarely shows distances similar to those belonging to the raw fragments (Fig. 8d). In general, average Fe-O bond distances in C samples seem to be shorter than those of the A and B samples. The above-mentioned modifications can be ascribed to both a change in the Fe^2+^/Fe^3+^ ratio and to a variation of Fe coordination; unfortunately, in such complex systems, it is not possible to infer more detailed information about the effects of the process.

## Discussion

The results highlight an extreme variability of the trace elements composition, already attributed to an intrinsic consequence of the stone assemblages from CS, pigments and resins^19^. Nevertheless, PCA was able to sort out internal trends in the dataset structure, pointing out that:

1. A specific chemical contamination of the parent AS occurs depending on the wet or dry processing operated.
2. The dry polishing of AS induces a contamination determined by the working-tools composition (special steels)^37^;
3. The wet processed materials include several elements compatible with the ion content of the water; namely, the unexpectedly large Ca content observed in almost all the investigated wet samples is certainly linked to the water chemical composition. In fact, the wet processing involves recycled water from a closed circuit in all considered firms. The chemical signature of water is driven by the continuous contact with the abated sediment (which is only seldom removed).

The processing of AS results in detectable changes of the Fe speciation. Namely, the EXAFS analysis proves that, during both wet and dry processing, considerable amounts of metallic Fe (likely provided by working-tools) are added to the studied materials, identifiable by EPR as permanent magnetic phases. Moreover, by delving into the Fe-O bond distance, it is possible to notice that the modifications induced by the mechanical treatments extend to all the detected Fe forms by modifying Fe coordination and/or Fe oxidation state.

The process of dust contamination verified by both XRF and XAS analyses has to be considered as extremely relevant for toxicity studies: Pavan *et al*.^19^, in fact, have already pointed out that the presence of numerous redox active transition species coupled to RCS during respiration can have a relevant role in modulating (and especially improving) reactivity in HO˙ generation. The present data confirm that hetero-ions are provided to the final respirable dusts by the processing itself, and that difference can also occur between the wet and dry processing. Accordingly, this evidence can be linked to the high and variable toxicity of these dusts.

The AS processing is also effective in changing the radical speciation. At the initial stage are observed only h_Al_, radiogenic radicals of quartz, and resin radicals, these latter being very diluted. The manufacturing process does not annihilate h_Al_, but it generates new radicals (R and X), not observed in previous studies on CS bearing materials. These radicals are specifically linked to the quartz presence/absence and not to the type of processing (wet/dry). ESEEM shows that only weak dipolar interactions with H nuclei are operative, suggesting that R and X radicals are located neither in the inorganic portion, nor in the resin, but rather at their interface.

Our data suggest that the operating temperature during the process is confined in the range between 260 °C and 380 °C (values at which the Ti and h_Al_ centres are annihilated, respectively)^35^. This range compares well with simulations and experimental determinations reported by several authors^38–41^, and is lower than the degradation temperature of most polyester resins^42,43^. Unfortunately, we have not enough spectroscopic information to extend this consideration to the wet process too. Nevertheless, similar, or lower, T values can be expected. This information allows establishing that the resins effectively persist^19^, but they do not react chemically to form additional radical species.

Thus, we point out that the main role played by resins is the protection from annihilation of the inorganic radicals here described. The fact that radical species appear protected, in full agreement with the findings of Pavan *et al*.^19^, confirms the anomalous behaviour of the interaction between silica dusts arising from AS, if compared with other conventional sources of exposure to RCS.

## Conclusion

The information provided by the present multianalytical investigation of artificial stone samples allows us to complete the results provided by Pavan *et al*.^19^. We confirm the extremely wide variability of the raw AS materials. The processed dusts clearly differ in the chemical signature from the parent AS, and they also differ in response to the operated dry/wet process. Moreover, significant differences in the Fe and radical speciation are observed between raw and processed materials. From these considerations, workers operating at different tasks may be exposed to dusts having different features, and the toxicity of the AS may be linked not only to internal properties but also to modifications externally induced by the processing. A specific task linked to the variable exposure for ASW is represented by the large amount of cristobalite, found in samples #4: this phase can play a confusing role in the evaluation of the degree of toxicity of the ASW cohorts^44^. The stability of the radicals observed in this study, and the persistence of the resin after the processing could suggest that resin, coating the RCS particles, is able to protect surface radicals for a limited time during the interaction with the lung lining fluids. This mechanism can thus be correlated to the higher toxicity of the AS powder with respect to the conventional silica bearing materials, whose unprotected radicals could be annihilated before reaching the lung tissues. Nevertheless, the fact that at least a part of the contaminants is available for the interaction with tissues immediately after the exposure (i.e. the metal contamination provided by the type of processing), while suggesting that complex paths of toxicity may arise, seems in line with the observation provided by Hoy *et al*.^1^, concerning the role of high level exposures during short periods in a working day.

## Supplementary information

Supporting Information

## References

[1] Hoy RF (2018). Artificial stone-associated silicosis: A rapidly emerging occupational lung disease. Occup. Environ. Med..

[2] Chang FC, Lee MY, Lo SL, Lin JD (2010). Artificial aggregate made from waste stone sludge and waste silt. J. Environ. Manage..

[3] Peng L, Qin S (2018). Mechanical behaviour and microstructure of an artificial stone slab prepared using a SiO_2_ waste crucible and quartz sand. Constr. Build. Mater..

[4] Lee MY (2008). Artificial stone slab production using waste glass, stone fragments and vacuum vibratory compaction. Cem. Concr. Compos..

[5] Hamoush S, Abu-Lebdeh T, Picornell M, Amer S (2011). Development of sustainable engineered stone cladding for toughness, durability, and energy conservation. Constr. Build. Mater..

[6] Gomes Ribeiro CE, Sanchez Rodriguez RJ, Carvalho EA (2017). Microstructure and mechanical properties of artificial marble. Constr. Build. Mater..

[7] Sarami N, Mahdavian L (2015). Effect of inorganic compound on artificial stones properties. Int. J. Ind. Chem..

[8] Sarami N, Mahdavian L (2017). Mechanical Properties of Artificial Stones Produced from Sludge of Stone-Cutting Factories (SSCF): The Effects of Nano-fillers (αTiO_2_ and ZnO Nanoparticles). Silicon..

[9] Santos EA (2015). Development of Epoxy Matrix Artificial Stone Incorporated with Sintering Residue from Steelmaking Industry. Mater. Res..

[10] Grubstein A (2016). Radiological evaluation of artificial stone silicosis outbreak: Emphasizing findings in lung transplant recipients. J. Comput. Assist. Tomogr..

[11] Paolucci V (2015). Silicosis in Workers Exposed to Artificial Quartz Conglomerates: Does It Differ From Chronic Simple Silicosis. Arch. Bronconeumol. English Ed..

[12] García Vadillo C, Gómez JS, Morillo JR (2011). Silicosis in Quartz Conglomerate Workers. Arch. Bronconeumol. English Ed..

[13] Martínez C (2010). Silicosis, una enfermedad con presente activo. Arch. Bronconeumol..

[14] Kramer MR (2012). CaesarStone silicosis: Disease resurgence among artificial stone workers. Chest..

[15] Rosengarten D (2017). Survival following lung transplantation for artificial stone silicosis relative to idiopathic pulmonary fibrosis. Am. J. Ind. Med..

[16] Shtraichman O (2015). Outbreak of autoimmune disease in silicosis linked to artificial stone. Occup. Med..

[17] Bartoli, D. *et al*. Silicosis in employees in the processing of kitchen, bar and shop countertops made from quartz resin composite. Provisional results of the environmental and health survey conducted within the territory of USL 11 of Empoli in Tuscany among employees. *Ita. J. Occ. Environ. Hyg.* **3**, 138–143 (2012).

[18] International Agency for Research on Cancer, IARC Monographs: Arsenic, Metals, Fibres, and Dusts, 100C (2012).

[19] Pavan C (2016). Abrasion of artificial stones as a new cause of an ancient disease. Physicochemical features and cellular responses, Toxicol. Sci..

[20] Mishali M, Weiler D (2017). Psychological factors causing nonadherence to safety regulations in Israel’s stone and marble fabrication industry: Unveiling the source of worker noncompliance, Cogent Bus. Manag..

[21] d'Acapito Francesco, Lepore Giovanni Orazio, Puri Alessandro, Laloni Alessio, La Manna Fabrizio, Dettona Eric, De Luisa Aleksander, Martin Andrea (2019). The LISA beamline at ESRF. Journal of Synchrotron Radiation.

[22] Mathon O (2015). The time-resolved and extreme conditions XAS (Texas) facility at the European Synchrotron Radiation Facility: The general-purpose EXAFS bending-magnet beamline BM23. J. Synchrotron Radiat..

[23] Aitchison, J. *The Statistical Analysis of Compositional Data*, (Springer Netherlands, Dordrecht 1986).

[24] Varmuza, K. & Filzmoser, P. *Introduction to Multivariate Statistical Analysis in Chemometrics*, CRC Press, Taylor & Francis Group (2009).

[25] Buccianti A, Grunsky E (2014). Compositional data analysis in geochemistry: Are we sure to see what really occurs during natural processes?. J. Geochemical Explor..

[26] Burdenski, T. K. J. Evaluating Univariate, Bivariate, and Multivariate Normality Using Graphical Procedures (2000).

[27] Ramzan S, Zahid FM, Ramzan S (2013). Evaluating Multivariate Normality: A Graphical Approach, Middle-East. J. Sci. Res..

[28] Kaufman, L. & Rousseeuw, P. J. Wiley InterScience (Online service), Finding groups in data: an introduction to cluster analysis, Wiley (1990).

[29] Aitchison J, Greenacre M (2002). Biplots of Compositional Data. J. R. Stat. Soc. Ser. C (Applied Stat.).

[30] Carbone C (2008). Multifrequency EMR and magnetic characterization of synthetic powdered hematite. J. Phys. Chem. C..

[31] Piligkos S, Laursen I, Morgenstjerne A, Weihe H (2007). Sign and magnitude of spin Hamiltonian parameters for Mn_2+_ impurities in calcite. A multi- and low-frequency EPR study. Mol. Phys..

[32] Anthony, J. W., Bideaux, R. A., Bladh, K. W. & Nichols, M. C. ed., Handbook of Mineralogy, Mineralogical Society of America, Chantilly, VA 20151-1110, USA, n.d., http://www.handbookofmineralogy.org/.

[33] Romanelli M (2012). ESEEM of industrial quartz powders: insights into crystal chemistry of Al defects. Phys. Chem. Miner..

[34] Sergei, Y. T. & Dikanov, A. *Electron Spin Echo Envelope Modulation (ESEEM) Spectroscopy*. (CRC Press, Boca Raton 1992).

[35] Ikeya, M. ESR Dosimetry: Dosimeter, Accident Dose and Foodstuffs, in: New Appl. Electron Spin Reson., World Scientific, 395–426 pp (1993).

[36] Di Benedetto F (2014). Variability of the health effects of crystalline silica: Fe speciation in industrial quartz reagents and suspended dusts-insights from XAS spectroscopy. Phys. Chem. Miner..

[37] DeGarmo, E. P., Black, J. T. & Kohser, R. A. DeGarmo’s Materials and Processes in Manufacturing, Tenth ed., Wiley, New York (2007).

[38] Xu XP, Huang H, Zeng WM (2003). Thermal Study of Rock Grinding, Part 2: Temperature Analysis. Key Eng. Mater..

[39] Zhan YJ, Xu XP (2012). An experimental investigation of temperatures and energy partition in grinding of cemented carbide with a brazed diamond wheel. Int. J. Adv. Manuf. Technol..

[40] Irša J, Galybin AN (2009). Heat flux reconstruction in the grinding process from temperature data. WIT Trans. Modelling Simul..

[41] Brosse A, Naisson P, Hamdi H, Bergheau JM (2008). Temperature measurement and heat flux characterization in grinding using thermography. J. Mater. Process. Technol..

[42] Bansal RK, Mittal J (1989). Thermal Stability and Degradation Studies of Polyester Resins. J. App. Pol. Sci..

[43] Muralidhara KS, Sreenivasan S (2010). Thermal degradation kinetic data of polyesteR, cotton and polyestercotton blended textile material. World Appl. Sci. J..

[44] Nattrass C, Horwell CJ, Damby DE, Brown D, Stone V (2017). The effect of aluminium and sodium impurities on the *in vitro* toxicity and pro-inflammatory potential of cristobalite. Environ. Res..

[45] Sevillano E, Meuth H, Rehr JJ (1979). Extended X-ray absorption fine structure Debye-Waller factors. I. Monatomic crystals. Phys. Rev. B.

