# Supplementary material for: Chemical variability of artificial stone powders in relation to their health effects
Source: Sci Rep. 2019 Apr 25;9:6531. doi: 10.1038/s41598-019-42238-2 (PMC6484096; doi:10.1038/s41598-019-42238-2)
Supplement: Supplementary file 1 — Supporting Information [file 41598_2019_42238_MOESM1_ESM.docx]

***Supporting information***

**to**

**Chemical variability of artificial stone powders in relation to their health effects**

Francesco Di Benedetto^1,2,*^, Andrea Giaccherini^1,2^, Giordano Montegrossi^2,3^, Luca A. Pardi^4^, Alfonso Zoleo^5^, Ferdinando Capolupo^6^, Massimo Innocenti^2,6^, Giovanni O. Lepore^7^, Francesco d’Acapito^7^, Fabio Capacci^8^, Carla Poli^8^, Tonina Enza Iaia^8^, Antonella Buccianti^1,3^ and Maurizio Romanelli^1^

1 – Dipartimento di Scienze della Terra, Università degli Studi di Firenze, via La Pira 4, Firenze, Italy

2 – INSTM Research Unit of Florence, Firenze, Italy

3 – CNR – Istituto di Geoscienze e Georisorse, via La Pira 4, Firenze, Italy

4 - CNR – Istituto dei Processi Chimico Fisici, via Moruzzi 1, Pisa, Italy

5 – Dipartimento di Scienze Chimiche – Università di Padova, Via Marzolo 1, Padova, Italy

6 – Dipartimento di Chimica, Università degli Studi di Firenze, via della Lastruccia 3, Sesto Fiorentino, Italy

7 – CNR – Istituto di Officina dei Materiali - OGG c/o, ESRF, Grenoble, France

8 - Dipartimento di Prevenzione, PISLL, Health Agency of Tuscany (USL Toscana Centro)

* - corresponding author: francesco.dibenedetto@unifi.it

**Section A - Investigated samples**


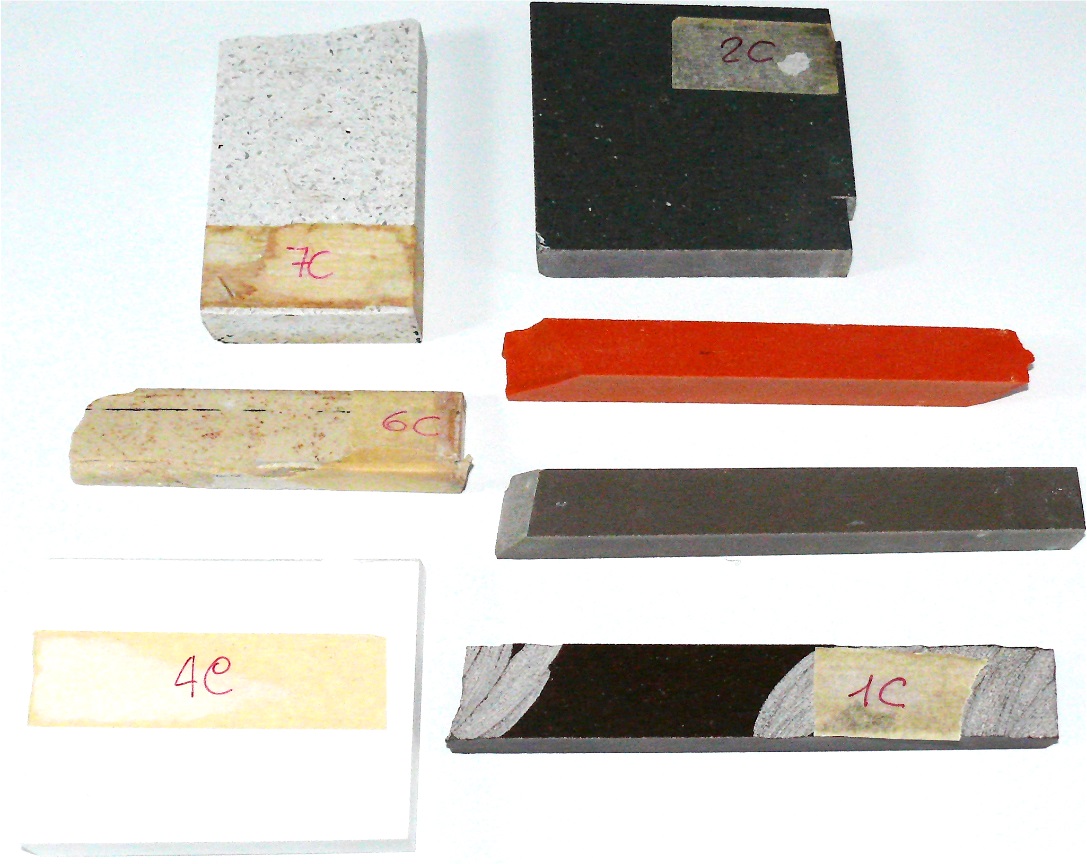


**5C**

**3C**

Photographs of the #C samples investigated in this study.

The cutting under wet conditions has been operated through a disk saw on all samples of the #A series but 2A, for which a pantograph cut was operated. Wet samples were maintained in their original environment until the analysis, then let dry at the air, under fume hood.

**Section B Details of the experimental procedures**

*SEM/EDS*

Aliquots of the samples were placed on stubs using double-sided conductive adhesive tape and coated with graphite. Secondary electron micrographs were obtained using a SEM ZEISS EVO MA15 (at the MEMA – Centro di Servizi di Microscopia Elettronica e Microanalisi, University of Florence) with accelerating voltage of 20 KV. Additional micrographs were obtained at the Microimaging Lab of ESRF (Grenoble, France), through a LEO 1530 scanning electron microscope (SEM), equipped with a Schottky-type field-emission electron source.

*XRF*

XRF measurements were performed with WD-XRF Rigaku PrimusII (CRIST – Structural Crystallography Interdepartmental Center, University of Florence), equipped with a Rh X-ray source and Silicon monochromator. The diameter of the X-ray spot suitable for measurements ranges from 0.5 to 40 mm, and this capability was used to focus on the different samples: all samples belonging to the #A and #B series were prepared as 2 inches (5.08 cm) pressed pellets, whereas those belonging to the #C series were investigated as thick sections (having a working surface area not greater than 1.5-2 cm^2^).

The pressed pellets were prepared by homogenizing 5 g of sample with ~ 1 g of binder (Spectroblend 44 μm powder; Chemplex Industries, inc.) and pressing them through uniaxial hydrostatic press. Conversely, the thick sections were realized from the original fragments of the #C samples, by cutting them in the approximate form of a parallelepiped, whose dimensions are ~20 x ~10 x ~0.3 mm.

The results of the so obtained XRF analysis are intrinsically semi-quantitative: a standardless procedure was adopted; under this procedure, a semi-quantitative matrix effect is evaluated by means of the SQX software (quantitative tool of the ZSX 3.5 package provided by Rigaku) through the comparison with internal libraries. The procedure is periodically calibrated by means of a set of opportune standards. No calibration curves for the considered elements were prepared, so that a quantitative determination of the matrix effect occurring in the 21 analysed samples was not available. This procedure doesn’t allow to express uncertainty values for all individual analyses, but repeated calibration procedures performed using a set of standards allowed to assess that the uncertainty of the method is usually within 1 %.

The resulting values for the trace elements were accepted only if higher than 3 times the minimum detection limits (mdl), provided by the software for the considered analyses. In the case of significantly different mdl arising from the analysis of different samples, acceptance was evaluated with respect to the less favorable mdl value. Analytical concentrations are provided as normalized wt%.

Representative panoramic and detail XRF spectra are shown in the Figure S2 (a and b)


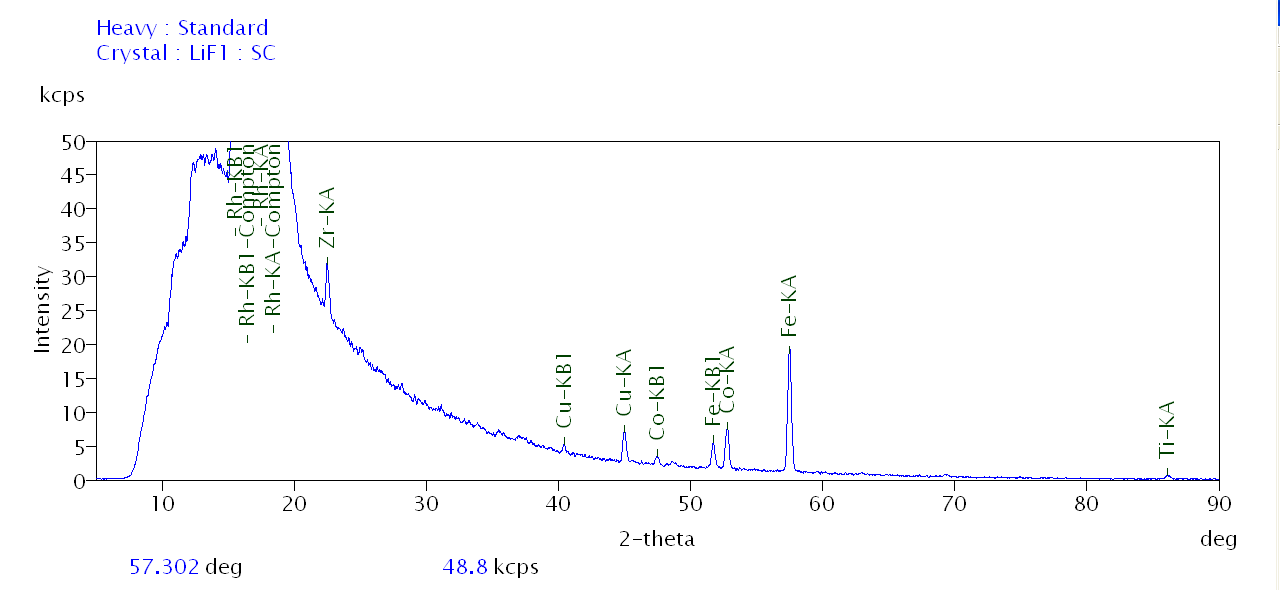
a


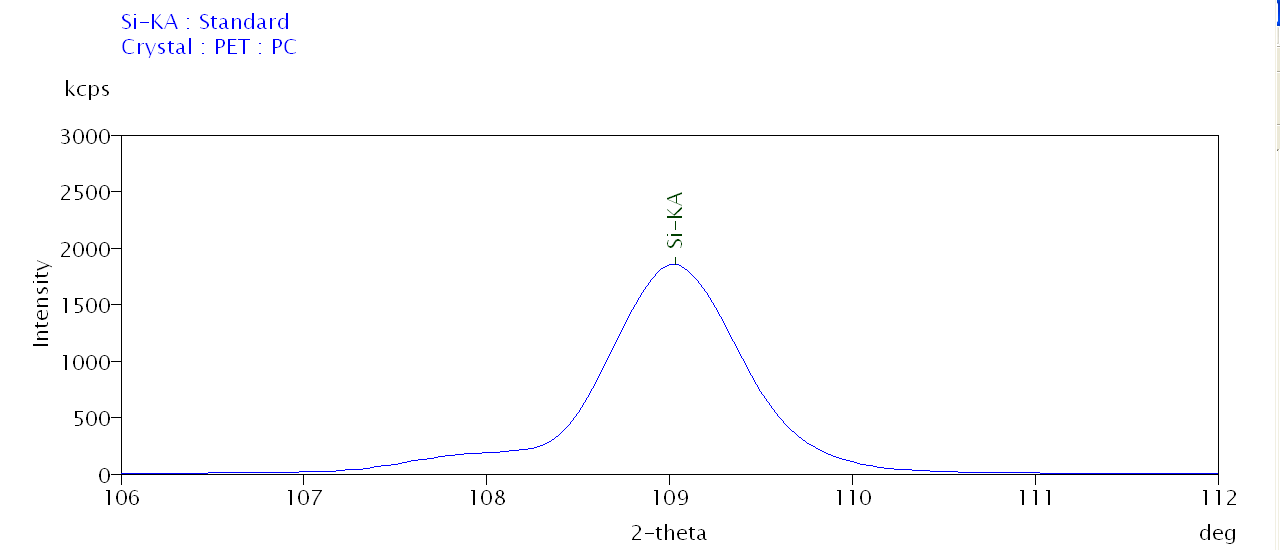
b

Figure S2 – a) Panoramic LIF XRF spectrum of the 1A sample, evidencing some trace elements; b) detail XRF spectrum of Si.

*XRPD*

XRPD patterns of the investigated samples were performed using a Bruker New D8 Da Vinci powder diffractometer (CRIST – University of Florence) and employing Ni-filtered Cu Kα (1.54187 Å) radiation. XRPD patterns were registered at 1,600 W (i.e. 40 kV, 40 mA) with a fast multi-channel detector in the 2θ range 3-80° applying a step size of 0.03° 2θ. XRPD data were refined by means of full-profile Rietveld algorithm, using the Fullprof software [**SI1**]. The same pellets prepared for the XRF were investigated through XRPD without further manipulation. The experimental XRD patterns are shown in the Figure S3. The main reflections pertain to quartz (samples 3A, 5A, 6A, 6B and 7B) and cristobalite (4A, 4B).





Figure S3 – experimental XRD patterns of selected investigated samples.

*EPR, EchoEPR, ESEEM experimental procedures*

EPR measurements were performed on a Bruker 200D spectrometer operating at X-band (ν ~9.5 GHz) equipped with ST100/700 variable-temperature assembly. The instrument is interfaced with Stelar software to a personal computer, for data acquisition and handling. The samples were measured as loose powders in polytetrafluoroethylene bags. Spectra were registered at room temperature. The calibration of the *g*-values was accomplished with reference to the DPPH [2,2-diphenyl-1-picrylhydrazyl] standard value (*g* = 2.0037). Repeated measurements assured the correct evaluation of the *g*-values. Spectra were registered in the magnetic field range 0-1000 mT (panoramic spectra) and 320-360 mT (detail spectra), with 0.5 G modulation amplitude and 100 kHz modulation frequency. Nominal resolution and scan speed were set to 0.48 mT / 5 mT∙s^-1^ and to 0.02 mT / 0.2 mT∙s^-1^ for the panoramic and detail spectra, respectively.

Cw-EPR measurements were also carried out at 35 K, to improve the spectral quality and to perform the spectral attribution of the radical species, by using a Bruker ELEXYS X-band EPR, equipped with an Oxford CF935 cryostat and a dielectric cavity with high Quality (Q) factor.

The study of the radical species associated to the investigated samples was carried out through the combined use of detailed c.w. EPR spectra, carried out at 35 K, and of EEPR spectra. These latter were chosen to obtain an increased selectivity towards a single species, due to the different behaviour of different species in the time domain.

Pulsed EPR investigations included the registration of both field swept Echo-EPR and Electron Spin Echo (ESE) decays. All measurements were carried out at 35 K on the same Bruker ELEXYS X-band EPR. Echo-EPR spectra were registered after a two-pulse π/2-τ-π-τ sequence at operating frequency of ~9.70 GHz. Magnetic field was varied in the range 300-360 mT. ESE decays were registered after a three-pulse π/2-τ-π/2-T-π/2-τ sequence, setting the magnetic field at the value corresponding to the maximum intensity of the Echo-EPR spectrum of the same sample. Experimental time domain was in the range 0-2140 ns, with a time step of 4 ns and a dead-time of 96 ns.

Electron Spin Echo Envelope Modulation (ESEEM) patterns of selected samples (i.e. 3a, 4a, 5a, 2b, 3b, 4b, 5b) were extracted by subtraction of a decay function obtained by refining a biexponential model, the form of which was y = Σ_i_A_i_e^−B(i)x^. The resulting nuclear modulation pattern was performed, to sort out the main frequency patterns: Fourier Transforms (FT) of the ESEEM’s were obtained by Hanning windowing and zero-filling procedures.

*XAS*

X-ray Absorption Spectroscopy (XAS) measurements at the Fe-*K* edge (7112 eV) were performed during two different experimental sessions at BM-23 [**22**] and LISA beamlines (BM-08 [**21**]) at the European Synchrotron Radiation Facility (ESRF, Grenoble – France).

Spectra were measured in fluorescence mode by means of a 12- and 13-elements solid state (high purity Germanium) detector for BM08 and BM23, respectively. A Fe reference foil was placed after the sample chamber, allowing to acquire a spectrum simultaneously with each measurement on the samples and thus accurately calibrate the energy. This was not possible for the #C samples, where the sample thickness prevented the X-ray beam from being transmitted through the sample. For all samples, detailed spectra were acquired both in the X-ray absorption near-edge structure (XANES) and the EXAFS regions. The software ATHENA [**SI2**] was used to calibrate energy (eV) and to average multiple spectra. Standard procedures [**SI3**] were followed to extract the structural EXAFS signal (*k*χ(*k*)): pre-edge background removal, spline modelling of bare atomic background, edge step normalization, and energy calibration. Model atomic clusters centred on the absorber atom were obtained by ATOMS [**SI4**]. EXAFS spectra were fitted through the ARTEMIS software [**SI2**] in the Fourier-Transform (FT) space; theoretical amplitude and phase functions were generated using the FEFF8 code [**SI5**].

**Section C Details of the statistical analysis of the XRF compositional data**

*Cluster analysis*

To avoid artificial constrain [**SI6**], compositional data, i.e. data including only relative information, need to be transformed before to apply the standard discriminant analysis methods that are designed for the Euclidean space. Thus, a cluster analysis algorithm was applied with the aim to point out the presence of groups characterized by internal compositional similarity: these groups can be considered “natural”, so they can be related to significant chemical changes among raw and processed samples.

Data were transformed by using the log-centred transformation [**23**]:


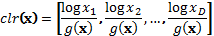


where *x_i_* are the *D* variables of the composition and *g*(**x**) is the row-wise geometric mean


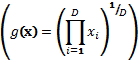

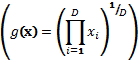


The metric used to evaluate the distance among samples was the squared Euclidean distance while cases were linked by using the Ward criterion [**28**]. Data below the detection limits were substituted by using the Bayesian multiplicative approach for compositional data proposed by Martin-Fernandez et al. [**SI7**].

*Biplot analysis*

The biplot is a widely and powerful tool used with multidimensional data sets to describe and display the simultaneous relationships between observations and variables [**29**]. Let **x** be the matrix of log-centred transformed data of dimension *n* x *D,* where usually the *n* rows represent observations and the *D* columns the log-centred variables. The main idea of biplot is to represent, exactly, by means of two sets of vectors the rows and the columns of any matrix whose rank is 2 [**SI8**].

If the whole variance-covariance structure of the matrix is significant, on a binary plot it is possible to recognize the whole relationships among samples and variables. The two axes of the biplot represent the direction where, in the multivariate space, variability is better explained in decreasing order. In our case, the biplot shown in Figure 3 (see article) is able to explain about 60% of the data variability; if the limited number of cases is taken into account, the obtained result appears to be considerable.

The mean features of a biplot useful to interpret the variance-covariance structure of a data set are given by:

1. an origin *O* which represents the centre of the compositional dataset;
2. a vertex at position **h_i_** for each of the *D* parts (variables clr-transformed);
3. a case marker at positioning **g_j_** for each of the n samples or cases.

The join of *O* to a vertex **h_i_** is termed 'ray' while the join of two vertices **h_i_** and **h_j_** is a 'link'. In this context, the length of a ray is related to the variance of the related log-centred variable, while the length of the link between two vertices inform us about the variance of the log-ratio between the variables associated to the two rays. Cosines of the angles between links estimate correlations between log-ratios and if two links are at right angles, zero correlation of the two log-ratios is expected. If vertices *i* and *k* coincide, or nearly so, this means that $var\left[ log\left( {x_{i}}/{x_{k}} \right) \right]$is about zero and the ratio ${x_{i}}/{x_{k}}$ is constant. If a subset of vertices is collinear, the associated sub-composition has unidimensional variability and a strong internal relationship characterizes the variables.

*Ternary diagrams*

As stated in the text, ternary diagrams including selected subcompositions (i.e. triads of elements chosen on the basis of their variability in the biplot, or to test specific hypotheses) were used. For a better visualization, the samples in these plots were centred so that the barycentre of the dataset was located in that of the triangle and all the observations followed the same translation. PC1 and PC2 (red and yellow curves, where present) are the two main directions explaining, in decreasing order, the data variability.

**Section D – Mineralogical and morphological characterization of the sample**

Figure D depicts a representative example of the difference in morphology between the by-product of the industrial process (the powders from the mechanical treatment) and the parent materials (the original synthetic stone). The synthetic stone (sample 5C), as it is, has plain surface without apparent detectable micro-particles on it, with the main features in the size range of 200-300 μm, immersed in a very fine matrix. The by-product of the industrial process is a powder consisting of irregularly shaped micro-particles ranging within three distinct size intervals: 200-100 μm, 50-10 μm and 2-0.5 μm. Apparently, the last range is the largely most frequent while the bigger particles rarely occur.


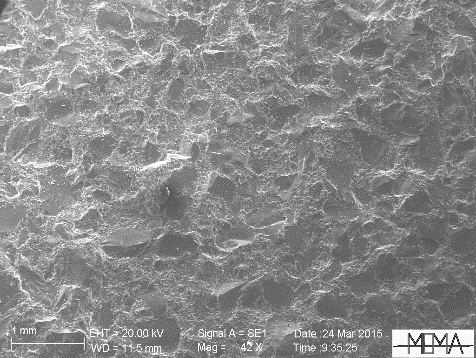

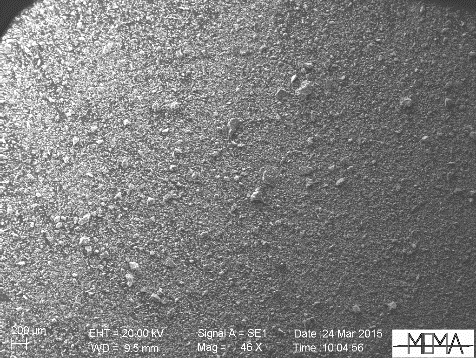

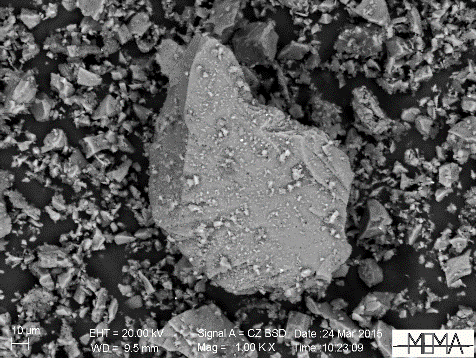


**a**

**b**

**c**

Figure D - Micrographs of the a) sample 5C at 42x b) sample 5B at 46x c) sample 5B at 1000x.

The powder XRD patterns of all the samples show an amorphous background probably due to the organic fraction of the material. In general, the XRD analysis reveals the clear difference between the base products and the byproducts of the industrial process. The prevalent presence of quartz in each sample is reckoned (up to 99%). In other cases, the amount is overwhelming for the samples C while for A and B different mineralogical phases have been found, reducing the amount of quartz down to 80%. Specifically, the samples A show a relevant calcite content.

Samples 4 represent three related outliers containing cristobalite (instead of quartz) and rutile as the most abundant phases. An uptake of calcite is observed in sample 4A, similar to what found for most of samples of the #B series. Minor uptakes (1-5 wt%) of illite, chlorite and, more frequently, feldspar have been seldom reckoned up.

**Section E XRF compositional data**

XRF compositional data, expressed as wt%. Uncertainties are assumed as ±1 on the last significant digit. mdl – minimum detection limit.

**Section F sub-composition Ca-Co-Ti**

The sub-composition Ca-Co-Ti was chosen as representative of the grouping in Figure 3, due to their ray lengths. However, the sample discrimination provided by these elements is not complete. This suggests that further processes are responsible of the compositional changes affecting groups A, B and C.


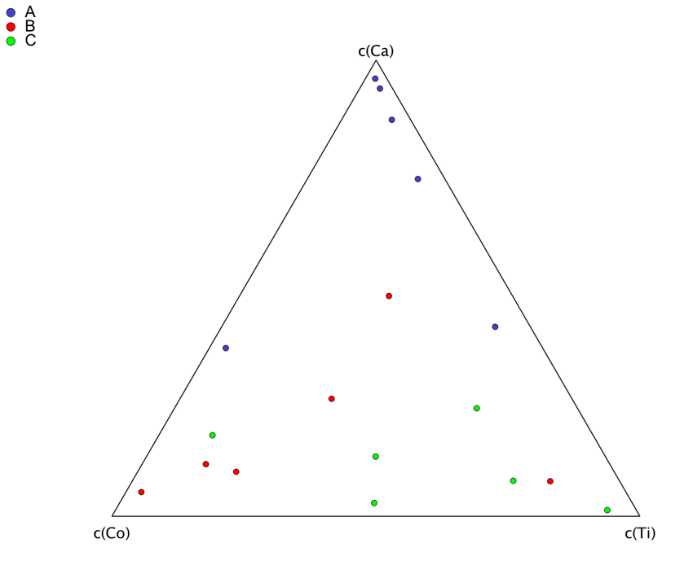


**Section G - Experimental cw EPR spectra of the investigated samples (A series), registered at 298 and 35 K. Spectral attribution is discussed in the text and listed in the Table 3.**



a

b



c

d



e

f



g

**Experimental cw EPR spectra of the investigated samples (B series), registered at 298 and 35 K. Spectral attribution is discussed in the text and listed in the Table 3.**



a

b



c

d



e

f



g

**Experimental cw EPR spectra of the investigated samples (G series), registered at 298 and 35 K. Spectral attribution is discussed in the text and listed in the Table 3.**



a

b



c

d



e

f



g

**Section H - Detailed Experimental cw EPR spectra of the investigated samples (A, B and C series), registered at 35 K. Spectral attribution is discussed in the text.**



**#A series**



**#B series**



**#C series**

**Section I - FT of the ESEEM patterns of the investigated samples. Spectral attribution is discussed in the text.**

**



**










**Section J - Summary of the species identified by LCF.**

|  | **Fe^0^** | **Gth** | **Hrc** | **Mgn** | **Amt** | **Mgm** | **Wst** | **Flt** | **Hmt** | **Frd** | **R factor*** |
| --- | --- | --- | --- | --- | --- | --- | --- | --- | --- | --- | --- |
| 1A | 0.1 | 0.8 | 0.1 |  |  |  |  |  |  |  | 0.0004 |
| 1B | 0.3 | 0.6 |  | 0.1 |  |  |  |  |  |  | 0.0005 |
| 1C |  | 0.8 | 0.1 |  |  | 0.1 |  |  |  |  | 0.0004 |
| 2A |  |  | 0.5 |  |  |  |  | 0.6 |  |  | 0.002 |
| 2B | 0.7 |  |  | 0.1 |  |  |  |  | 0.2 |  | 0.0004 |
| 2C | 0.1 |  |  | 0.6 |  | 0.3 |  |  |  |  | 0.0004 |
| 3A |  |  |  |  |  |  |  |  |  |  |  |
| 3B | 0.4 |  |  | 0.3 |  |  | 0.3 |  |  |  | 0.002 |
| 3C |  |  |  |  |  |  |  |  |  |  |  |
| 4A | 0.5 |  |  | 0.4 |  |  |  | 0.1 |  |  | 0.0008 |
| 4B | 0.6 | 0.1 |  |  | 0.3 |  |  |  |  |  | 0.0006 |
| 4C | 0.1 |  |  |  | 0.6 | 0.3 |  |  |  |  | 0.004 |
| 5A |  |  | 0.4 | 0.2 |  |  |  | 0.4 |  |  | 0.0007 |
| 5B | 0.1 | 0.6 |  | 0.3 |  |  |  |  |  |  | 0.0003 |
| 5C |  | 0.8 | 0.1 |  |  | 0.2 |  |  |  |  | 0.0006 |
| 6A | 0.1 | 0.4 |  | 0.5 |  |  |  |  |  |  | 0.0006 |
| 6B | 0.1 | 0.5 |  |  |  | 0.4 |  |  |  |  | 0.002 |
| 6C |  | 0.7 | 0.1 |  |  | 0.3 |  |  |  |  | 0.002 |
| 7A | 0.3 |  |  | 0.6 |  |  |  | 0.1 |  |  | 0.0005 |
| 7B | 0.2 |  | 0.1 |  |  |  |  |  |  | 0.6 | 0.0005 |
| 7C |  |  |  |  | 0.5 | 0.2 | 0.3 |  |  |  | 0.001 |

Note: standards used - goethite (Gth), hercynite (Hrc), magnetite (Mgn), Fe-bearing amethyst (Amt.), maghemite (Mgm), wustite (Wst), fayalite (Flt), hematite (Hmt), ferrihydrite (Frd). *Σ(data-fit)^2^/Σ(data)^2^

**Representative LCF in the XANES region for samples 6A, 6B and 6C**





**References (not included in the main article)**

SI1 - Rodriguez-Carvajal J (1993). Phys B 192:55–69

SI2 - Ravel B and Newville M (2005). J Synchrotron Radiat 12:537-541

SI3 - Lee PA, Citrin PH, Eisenberger PT, Kincaid BM (1981). Rev Mod Phys 53:769-806

SI4 - Ravel B (2001). J Synchrotron Radiat 8:314-316

SI5 - Ankudinov AL, Ravel B, Rehr JJ, Conradson SD (1998). Phys. Rev. B 58:7565-7576

SI6 - Buccianti A, Zuo R (2016). Appl Geochemistry 75:189–199

SI7 - Martín-Fernández J-A, Hron K, Templ M, Filzmoser P, Palarea-Albaladejo J (2015). Stat Model An Int J 15:134–158

SI8 - Gabriel KR (1971). Biometrika 58:453
